# ZnO-Controlled Growth of Monolayer WS_2_ through Chemical Vapor Deposition

**DOI:** 10.3390/ma12121883

**Published:** 2019-06-12

**Authors:** Zhuhua Xu, Yanfei Lv, Feng Huang, Cong Zhao, Shichao Zhao, Guodan Wei

**Affiliations:** 1 College of Materials & Environmental Engineering, Hangzhou Dianzi University, Hangzhou 310018, China; zhuhuaxu@hdu.edu.cn (Z.X.); grafengh@hdu.edu.cn (F.H.); zhaoshichao@hdu.edu.cn (S.Z.); 2 Tsinghua-Berkeley Shenzhen Institute (TBSI), Tsinghua University, Shenzhen 518055, China; zhao-c18@mails.tsinghua.edu.cn

**Keywords:** monolayer WS_2_, ZnO, CVD, controlled growth

## Abstract

Monolayer tungsten disulfide (2D WS_2_) films have attracted tremendous interest due to their unique electronic and optoelectronic properties. However, the controlled growth of monolayer WS_2_ is still challenging. In this paper, we report a novel method to grow WS_2_ through chemical vapor deposition (CVD) with ZnO crystalline whisker as a growth promoter, where partially evaporated WS_2_ reacts with ZnO to form ZnWO_4_ by-product. As a result, a depletion region of W atoms and S-rich region is formed which is favorable for subsequent monolayer growth of WS_2_, selectively positioned on the silicon oxide substrate after the CVD growth.

## 1. Introduction

Two dimensional (2D) materials such as graphene, hexagonal boron nitride, and transition metal dichalcogenides have attracted tremendous attention from scientists in materials science, physics, and chemistry for their monolayer structure and properties [1,2,3]. Monolayer WS_2_ is one of the typical 2D materials with a suitable direct band gap ca 2.0 eV showing potential applications in sensors, electronics, and optoelectronics [4,5,6,7,8]. Recently, many methods have been used to prepare monolayer WS_2_, such as mechanical exfoliation, as well as wet chemical and hydrothermal synthesis [9,10,11,12]. Chemical vapor deposition (CVD) is considered the most suitable method for the preparation of monolayer WS_2_ used for thin film devices [13]. In the CVD method, tungsten oxide and sulfide powders are commonly used as precursors, which evaporate at high temperature and react to form WS_2_. Large-scale monolayer WS_2_ has been reported to be grown on an Au substrate [14]. To integrate with silicon integrated circuit technology, the growth of WS_2_ on a silicon dioxide (SiO_2_) substrate is preferred [15,16]. Many works have focused on the deposition of the monolayer WS_2_ on the SiO_2_ substrate by CVD [17,18,19]. However, the deposition has poor reproducibility caused by the growth conditions [20]. In addition, the 2D WS_2_ obtained is combined with monolayer and multilayers [19,21,22]. The selective growth and the positioning of monolayer WS_2_ are not under control. Besides the controlled growth, the mechanism of the monolayer WS_2_ formation is still under discussion. Fan et al. provided an understanding of the dislocation-driven growth mechanism of 2D nanostructures in their work [23]. Cain et al. found that transition metal dichalcogenide monolayer growth proceeds from nominal lyoxi-chalcogenide nanoparticles which act as heterogeneous nucleation sites for monolayer growth [24]. The reaction of tungsten oxide in sulfur vapor suggests that the growth of WS_2_ is thermodynamically correlated with the sulfur concentration. We hypothesized that if the sulfur concentration could be controlled, it would be possible to deposit WS_2_ with the desired thickness.

Herein, we report a novel method to selectively grow monolayer WS_2_ on SiO_2_ substrate. ZnO crystal whisker is used to mediate the spacial distribution of sulfur concentration. We show that monolayer WS_2_ symmetrically distributes on both sides of the ZnO crystal whisker. By constructing a concentration distribution model, the monolayer growth mechanism can be discussed.

## 2. Materials and Methods

### 2.1. WS_2_ Monolayer Preparation

**Figure 1:**
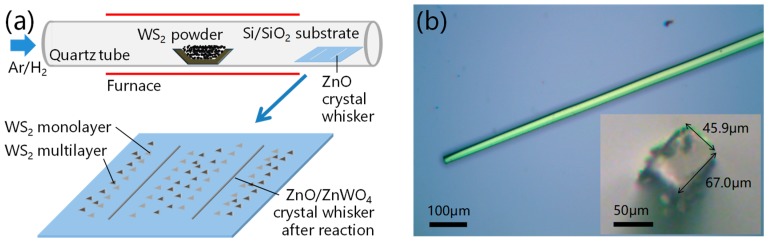
(**a**) Schematic diagram of the home-built chemical vapor deposition (CVD) system and tungsten disulfide (WS_2_) after CVD growth. (**b**) Optical microscopy image of ZnO crystal whisker transferred onto the surface of SiO_2_/Si substrate before WS_2_ growth. Insert illustration: cross section of ZnO crystal whisker with a length and width of 67.0 μm and 45.9 μm, respectively.

Figure 1a shows a schematic diagram of the home-built CVD system. WS_2_ powders (2.0 g, purity 99.5%, Aladdin, Shanghai, China) used as precursor were loaded into a quartz boat and put at the center of a quartz tube (1 inch in diameter). Silicon wafer with a 300 nm thick oxide layer used as substrate (SiO_2_/Si) was placed at the low temperature region of the quartz tube downstream of the carrier gas. Ar/H_2_ (5% H_2_) was used as carrier gas. ZnO crystal whiskers (1.5 cm in length) were obtained by thermal evaporation of ZnO powders according to the reference and transferred onto the substrate [25]. Figure 1b shows a typical ZnO crystal whisker with a size of 67.0 μm × 45.9 μm × 1500 μm.

For the growth of the WS_2_, the carrier gas with 35 sccm was introduced into the CVD system, which was evacuated to 70 torr. Then the furnace was heated from room-temperature to 1000 °C in 40 min and kept for 1 h. After that, the furnace was cooled down from 1000 °C to room-temperature under carrier gas flow.

### 2.2. Characterizations

Optical and photoluminescence (PL) imagings were carried out on a Jiangnan MV3000 digital microscope (Nanjing Jiangnan Novel Optics Co., Ltd.; Nanjing, China). Scanning electron microscopy (SEM) was conducted on a field emission scanning electron microscope (FESEM, ULTRA 55, Zeiss, Heidenheim, Germany). Photoluminescence (PL) and Raman spectra were acquired on a home-built Raman system, consisting of an inverted microscope (Ti eclipse, Nikon, Tokyo, Japan), a Raman spectrometer (iHR320, Horiba, Kyoto, Japan) with CCD detector (Syncerity, Horiba, Kyoyo, Japan) and a semiconductor laser at 532 nm (Uniklasers, Glasgow, UK). All measurements were performed at room temperature.

## 3. Results and Discussion

**Figure 2:**
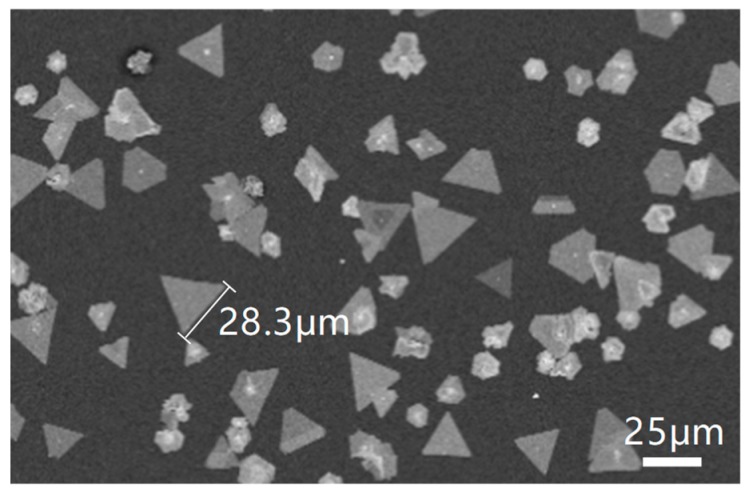
Scanning electron microscopy (SEM) image of WS_2_ grown on SiO_2_/Si substrate.

Figure 2 shows the SEM image of the WS_2_ grown on the SiO_2_/Si substrate. We can see the separated WS_2_ domains of a triangular and hexagonal shape. The maximum size of the domain is up to 28.3 μm.

**Figure 3:**
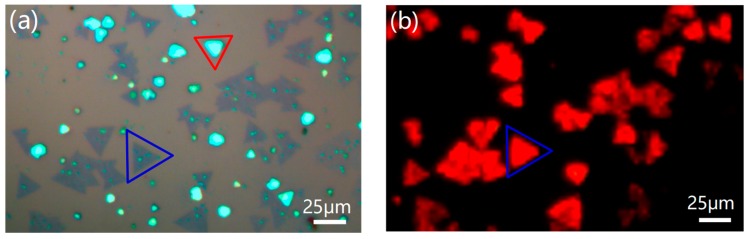
(**a**) Optical microscopy image of WS_2_ monolayer (marked with a blue triangle) and multilayer (marked with a red triangle). (**b**) The photoluminescence (PL) image corresponding to the sample (**a**). (**a**,**b**) are taken at the same location. The pattern with red color in (**b**) is due to the PL emission of monolayer WS_2_.

Figure 3a shows the optical microscopy image of the sample. The dark region marked with a blue triangle is monolayer WS_2_ while the bright region marked with a red triangle is due to multilayer WS_2_. The uniform color contrast of the monolayer indicates the thickness uniformity of the WS_2_ monolayer. Figure 3b shows the PL image corresponding to the sample in Figure 3a taken at the same location. The excitation wavelength was 485 nm. The monolayer WS_2_ displays super-bright red light emitting under irradiation. The patterns with the red color in Figure 3b remain as features of the monolayer WS_2_ domains in Figure 3a. However, the PL emission of multilayer WS_2_ is not strong enough to be detected by photoluminescence microscopy. The difference of the PL behavior between the monolayer and multilayer lies in the different electrical structures. Monolayer WS_2_ is a direct band gap, while multilayer is an indirect band gap [26]. The fluorescence quantum efficiency of the direct band gap semiconductor is much higher than that of the indirect [27,28]. Therefore, we only observe the PL image in the monolayer WS_2_.

**Figure 4:**
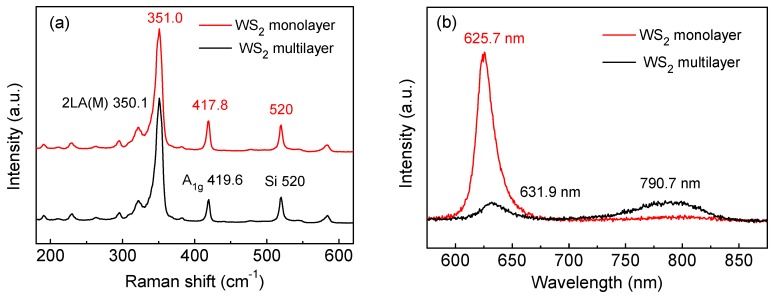
(**a**) Raman and (**b**) PL spectra of WS_2_ monolayer (red line) and multilayer (black line) corresponding to the WS_2_ domains in Figure 3a.

Figure 4 shows the Raman and PL spectra of the WS_2_ monolayer (red line) and multilayer (black line). Raman spectroscopy was used to identify the number of two dimensional material layers. Raman peaks at 351.1 cm^−1^, 417.2 cm^−1^ are the fingerprint peaks of monolayer WS_2_, which are due to the second-order longitudinal acoustic mode (2LA(M)), and out-of-plane vibration mode (A_1g_), respectively [29]. With the increase of the number of layers, the 2LA(M) peak red shifts and the A_1g_ peak blueshifts. The 2LA(M) and A_1g_ peaks are observed in Figure 4a. For the domain marked with a blue triangle in Figure 3a, Raman peaks located at 351.0 cm^−1^ (2LA(M)) and 417.8 cm^−1^ (A_1g_) were observed, indicating the thickness of the domain is monolayer. For the domain marked with a red triangle in Figure 3a, the 2LA(M) peak redshifted to 350 cm^−1^ and A_1g_ peak blueshifted to 419.6 cm^−1^, indicating the thickness of the domain is multilayer [27,29,30]. An intense PL emission peak at 625.7 nm was observed in Figure 3b, which is related to the direct band gap. Compared to the monolayer, the multilayer undergoes a transition from direct to indirect band gap resulting in a redshift of the PL peak and a sharp decrease of the PL intensity at 631.9 nm [27].

**Figure 5:**
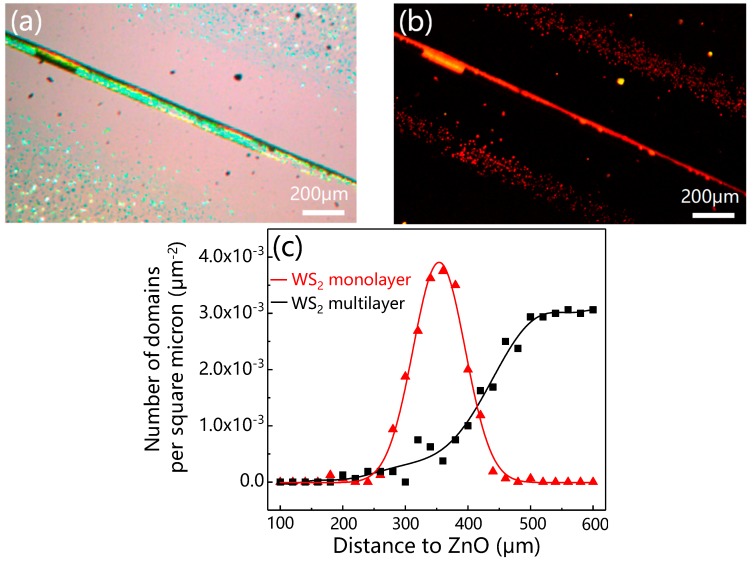
(**a**) The optical image of WS_2_ domains prepared with the assistance of ZnO whisker. (**b**) PL image corresponding to the image of (**a**). (**c**) WS_2_ domain distribution is a function of distance from the ZnO whisker. The origin is the ZnO crystal whisker. (**a**) and (**b**) were taken at the same location. The straight lines are related to ZnO whisker and the scale bars represent 200 μm.

Figure 5a,b shows the optical and PL image of WS_2_ grown near the region of ZnO whisker, respectively. To investigate the influence of ZnO crystal whisker on the growth of WS_2_ monolayer, we made distribution statistics of the WS_2_ monolayer and multilayer at one side of the ZnO crystal whisker. Figure 5c shows the distribution of statistical estimates of the WS_2_ domain density on the substrate. The direction of the horizontal axis is perpendicular to the growth axis of ZnO whisker. The coordinate of the ZnO whisker on the horizontal axis is zero.

WS_2_ domains hardly grew in the region (0–250 μm) close to the ZnO whisker. In the region (250–450 μm), a little farther away from the WS_2_ whisker, monolayer WS_2_ domains symmetrically grew on both sides of the ZnO crystal whisker, suggesting the ZnO crystal whisker played a crucial role in the growth of the WS_2_ monolayer. In the region (larger than 450 μm) far away from the ZnO whisker, where ZnO has hardly any impact on the growth of WS_2_, multilayer WS_2_ domains were observed. Therefore, monolayer and multilayers were grown separately in different zones of the substrate. To date, the position control of the WS_2_ monolayer has not been reported. The merit of our growth method lies in the accurate positioning of the WS_2_ monolayer using ZnO whisker.

**Figure 6:**
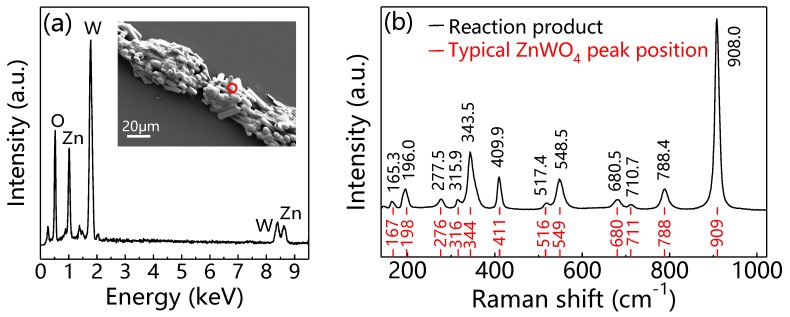
(**a**) EDS and (**b**) Raman spectra of ZnO crystal whisker after WS_2_ growth. Insert in (**a**) is the SEM image with a red circle where the EDS spectrum is taken from. The data in black and red color in (**b**) correspond to our experiment and reference [31,32], respectively.

Before analyzing the mechanism of monolayer WS_2_ growth in the presence of ZnO, we characterized the product of ZnO after WS_2_ growth by SEM, EDS, and Raman spectra. Besides oxygen and zinc, we found tungsten (W) element in the sample. The EDS spectrum intensity of W is even greater than that of Zn. In addition, the morphology transformed from the crystal whisker of ZnO to capsules as shown in the insert of Figure 6a. The results of EDS and SEM indicate that the chemical composition of the ZnO whisker may have been changed after the growth of WS_2_. Raman results further verified our assumption. Figure 6b is the Raman spectrum of ZnO crystal whisker after WS_2_ deposition. We find all the Raman peaks are due to ZnWO_4_. The slight difference of Raman peak position between our experiment and the reference may lie in the test conditions and/or strain in the sample. Therefore, ZnO transformed into ZnWO_4_ during the growth of WS_2_ domains.

At the high temperature of 1000 °C and low pressure of 70 torr, WS_2_ powder evaporated and decomposed into W and S atoms. According to the elementary composition of reaction product ZnWO_4_, W atoms reacted with ZnO and were consumed resulting in the depletion region of W atoms and the S-rich region around ZnO.

**Figure 7:**
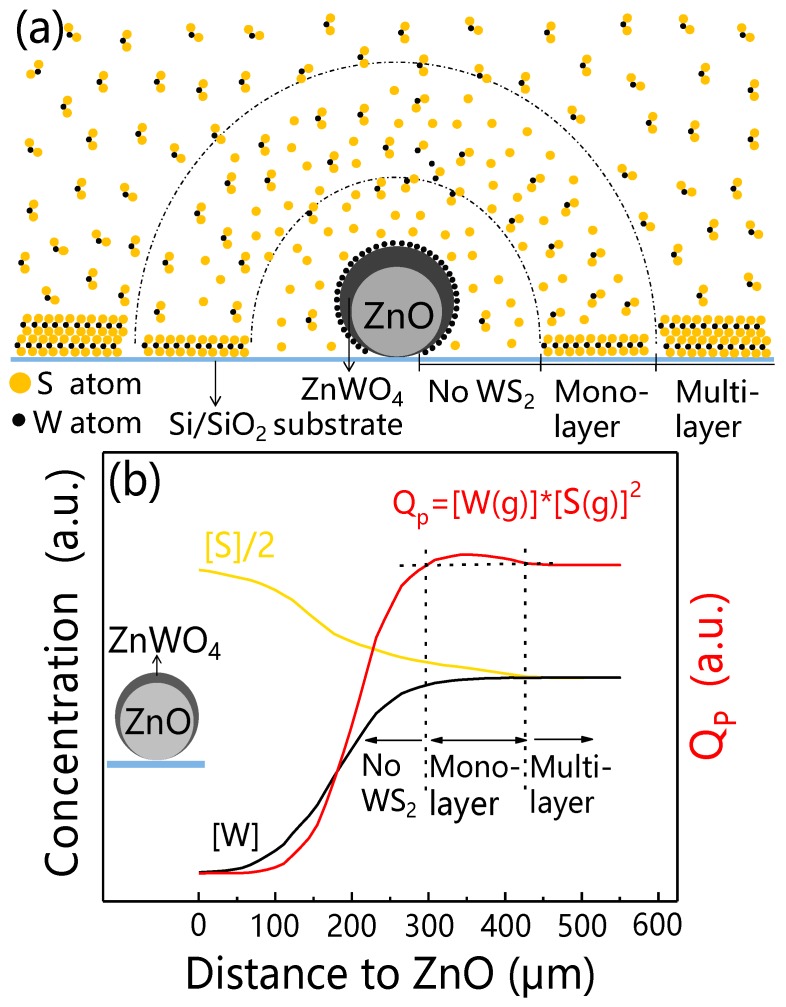
(**a**) Schematic diagram of W and S distribution around ZnO crystal whisker. (**b**) Distance dependence of W atom concentration, S atom concentration, and the reaction quotient (Q_P_). The direction of the horizontal axis is perpendicular to the growth axis of ZnO whisker. The coordinate of the ZnO whisker on the horizontal axis is zero. [W] and [S] represent W and S atom concentration in (**b**), respectively.

To discuss the growth mechanism, we built a distance dependent model of W and S distribution and WS_2_ domain growth. Figure 7a shows a schematic diagram of W and S distribution around ZnO crystal whisker. Figure 7b shows the distribution of estimates of W and S atoms concentration around the ZnO whisker. The direction of the horizontal axis is perpendicular to the growth axis of ZnO whisker. The coordinate of the ZnO whisker on the horizontal axis is zero. 

In the area far from the ZnO (larger than 450 μm), the growth of WS_2_ is barely influenced by ZnO. In this area, multilayer WS_2_ domains are grown. We consider the deposition of WS_2_ domains achieves chemical equilibrium. The equilibrium constant is denoted as K_C_.

In the area (0–450 μm) close to ZnO crystal whisker, W atoms reacted with ZnO to form ZnWO_4_ resulting in a decrease of the W atom concentration. WS_2_ precursors far from this area will diffuse to this region to keep the reaction running. With the reaction and diffusion of the WS_2_ precursor, W atoms are consumed and S atoms are accumulated in this area. With the distance increase, the W atom concentration increases and the S atom concentration decreases. They all reach equilibrium concentration when the distance is larger than 450 μm. Reaction quotient (Q_P_) increases first, and reaches the maximum value (250–450 μm), then decreases to the equilibrium constant K_C_. In the area of 0–250 μm to ZnO crystal whisker, the W atom concentration is low and the reaction quotient Q_P_ is no more than K_C_. Therefore, the WS_2_ domains hardly grow. In the area of 250–450 μm to ZnO crystal whisker, the larger Q_P_ (>K_C_) and S atom concentration promote the growth of monolayer WS_2_. The excess S atom is the key parameter for the monolayer growth.

## 4. Conclusions

In summary, we successfully prepared monolayer WS_2_ by a novel method. ZnO crystal whisker was used to position and promote the growth of WS_2_. The distribution statistics show monolayer WS_2_ was grown on both sides of the ZnO crystal whisker. By constructing a concentration distribution model, we were able to discuss the monolayer growth mechanism. The results reveal that gaseous sulfur and tungsten concentration are crucial for the thickness control of WS_2_. Higher concentration of sulfur and lower concentration of tungsten are of tremendous benefit for monolayer WS_2_ growth. This method would provide a way to grow and pattern monolayer WS_2_ and other two dimensional transition metal disulfides on silicon substrate for the fabrication of nano-optoelectronic devices.
